# Elevated Urinary Connective Tissue Growth Factor in Diabetic Nephropathy Is Caused by Local Production and Tubular Dysfunction

**DOI:** 10.1155/2015/539787

**Published:** 2015-06-15

**Authors:** Karin G. F. Gerritsen, Jan Willem Leeuwis, Maarten P. Koeners, Stephan J. L. Bakker, Willem van Oeveren, Jan Aten, Lise Tarnow, Peter Rossing, Jack F. M. Wetzels, Jaap A. Joles, Robbert Jan Kok, Roel Goldschmeding, Tri Q. Nguyen

**Affiliations:** ^1^Department of Pathology, University Medical Center Utrecht, 3584 CX Utrecht, Netherlands; ^2^Department of Nephrology and Hypertension, University Medical Center Utrecht, 3584 CX Utrecht, Netherlands; ^3^Department of Internal Medicine, University Medical Center Groningen, 9700 RB Groningen, Netherlands; ^4^HaemoScan, 9723 JC Groningen, Netherlands; ^5^Department of Pathology, Academic Medical Center, 1105 AZ Amsterdam, Netherlands; ^6^Steno Diabetes Center, 2820 Gentofte, Denmark; ^7^Department of Nephrology, Radboud University Nijmegen Medical Centre, 6525 GA Nijmegen, Netherlands; ^8^Utrecht Institute for Pharmaceutical Sciences, Utrecht University, 3584 CG Utrecht, Netherlands

## Abstract

Connective tissue growth factor (CTGF; CCN2) plays a role in the development of diabetic nephropathy (DN). Urinary CTGF (uCTGF) is elevated in DN patients and has been proposed as a biomarker for disease progression, but it is unknown which pathophysiological factors contribute to elevated uCTGF. We studied renal handling of CTGF by infusion of recombinant CTGF in diabetic mice. In addition, uCTGF was measured in type 1 DN patients and compared with glomerular and tubular dysfunction and damage markers. In diabetic mice, uCTGF was increased and fractional excretion (FE) of recombinant CTGF was substantially elevated indicating reduced tubular reabsorption. FE of recombinant CTGF correlated with excretion of endogenous CTGF. CTGF mRNA was mainly localized in glomeruli and medullary tubules. Comparison of FE of endogenous and recombinant CTGF indicated that 60% of uCTGF had a direct renal source, while 40% originated from plasma CTGF. In DN patients, uCTGF was independently associated with markers of proximal and distal tubular dysfunction and damage. In conclusion, uCTGF in DN is elevated as a result of both increased local production and reduced reabsorption due to tubular dysfunction. We submit that uCTGF is a biomarker reflecting both glomerular and tubulointerstitial hallmarks of diabetic kidney disease.

## 1. Introduction

Diabetic nephropathy (DN) is the leading cause of chronic kidney disease and the primary diagnosis in more than 40% of new patients on dialysis in several parts of the world including the United States [1]. Identifying the factors that contribute to the pathogenesis of DN is a critical step towards halting its progression. Connective tissue growth factor (CTGF; CCN2) is a matricellular protein involved in modulation of the extracellular environment and plays a role in the development and progression of diabetic complications [2–11]. In DN, increased renal CTGF expression has been described both in glomeruli and in the tubulointerstitium [4]. Urinary CTGF (uCTGF) is elevated in DN and correlates with markers of disease severity such as urinary albumin excretion and glomerular filtration rate (GFR) [12–14]. Furthermore, uCTGF has been shown to correlate with progression of microalbuminuria in diabetic patients [15]. Thus urinary CTGF might be suitable as a biomarker in monitoring DN. However, to interpret elevated uCTGF in diabetes, it is crucial to understand the mechanisms behind this elevation.

Both increased intrarenal production and elevated plasma CTGF have been suggested to account for elevated uCTGF in DN [13, 14]. We have shown in healthy volunteers and normoglycemic mice that blockade of proximal tubular reabsorption results in a major increase in uCTGF [16]. The aim of this study is to evaluate which factors contribute to elevated uCTGF in diabetes. In diabetic mice, we measured the fractional excretion (FE) of both endogenous CTGF and recombinant CTGF (i.e., not intrarenally derived CTGF) and examined the renal expression pattern of CTGF. Moreover, we examined the association between uCTGF and urinary markers in type 1 diabetic patients.

## 2. Materials and Methods

### 2.1. Animal Experiment

Diabetes was induced in nineteen 10–12-week-old male C57BL/6 mice (Harlan, Horst, Netherlands) by a single intraperitoneal injection of 200 mg/kg streptozotocin (STZ, Sigma, St. Louis, MO, USA) in sodium citrate buffer (100 mmol/L, pH 4.5). Blood glucose was determined one week after injection of STZ (MediSense Precision Xtra, Abbott, Abbott Park, IL, USA). Slow-release insulin pellets were implanted subcutaneously to stabilize the condition of the diabetic animals (LinBit, LinShin, Scarborough, ON, Canada). Eight control animals were injected with sodium citrate buffer. Ten weeks after STZ, renal function parameters and fractional excretion of CTGF were determined. Two miniosmotic pumps (model 1003D, ALZET, Cupertino, CA, USA, 100 *μ*L reservoir volume, release rate of 1 *μ*L/h) were implanted intraperitoneally under isoflurane anaesthesia. One pump was used for infusion of FITC-inulin. The other pump was used for simultaneous infusion of recombinant human CTGF (rCTGF, 38 pmol/h) in fourteen of the diabetic mice and four of the control mice. We used the proteolytic aminoterminal fragment of CTGF since this is the predominant form of CTGF detected in plasma and urine [13, 17–19]. To exclude that rCTGF had an effect on endogenous CTGF mRNA expression in the kidney, we infused four control mice and five diabetic mice with vehicle. The mice injected with rCTGF were used to determine FE of rCTGF and intrarenally derived uCTGF (see below).

Forty-eight hours after pump implantation, mice were put in metabolic cages overnight for timed urine collection. Plasma was collected before and after the urine collection, CTGF levels were determined, and time-weighted averages of plasma CTGF were calculated. All samples and metabolic cages were protected from light. Mice were killed 3 days after pump implantation and organs were harvested. All experiments were approved by the Animal Ethical Committee of the University of Utrecht and performed in accordance with national guidelines for the care and handling of animals.

### 2.2. Diabetic Patient Study

Three hundred and forty-nine well-characterized adult type 1 diabetic patients were selected from the outpatient clinic at Steno Diabetes Center (Copenhagen, Denmark). Forty-three of the patients with diabetic nephropathy had been previously analysed in a longitudinal study examining the impact of Losartan on uCTGF [12]. The study was approved by the Ethical Committee of Copenhagen County and performed in accordance with the Declaration of Helsinki. Demographic and clinical data were recorded, including age, sex, duration of diabetes, and body mass index. Creatinine was determined in venous blood samples using the Cobas Mira Plus (Roche, Basel, Switzerland). HbA_1c_ was determined using variant high-performance liquid chromatograph (Bio-Rad Laboratories, Hercules, CA, USA). The estimated GFR (eGFR) was calculated using the Modification of Diet in Renal Disease study method [20]. Albumin excretion rate (AER) was determined in 24 h urine collections using turbidimetry. Macroalbuminuria was defined as albuminuria >300 mg/24 h, microalbuminuria as albuminuria 30–300 mg/24 h, and normoalbuminuria as albuminuria <30 mg/24 h. Sixty patients were excluded from analysis because of incomplete data due to insufficient sample availability or incomplete patient characteristics.

### 2.3. CTGF Proteins, Antibodies, and ELISA

Recombinant human CTGFs and anti-CTGF antibodies were supplied by FibroGen Inc. (San Francisco, CA, USA). CTGF levels in plasma and urine were determined by sandwich ELISA, using specific antibodies (FibroGen) directed against distinct epitopes in the aminoterminal fragment of CTGF, detecting both full length CTGF and the N-fragment (N-CTGF), as described previously [16]. Two ELISA assays were used: an assay for detection of human or rCTGF in either human or mouse samples and an assay for detection of rodent CTGF in mouse samples. The antibody used for detection in the human CTGF assay does not cross-react with rodent CTGF and allows specific determination of rCTGF in mouse samples.

### 2.4. Laboratory Measurements (Patient Studies)

CTGF was determined as described above. Urinary *α*1-microglobulin (*α*1M) was measured by competition enzyme immunoassay. Maxisorp microtiter plates (Nunc, Rochester, NY, USA) were coated with 0.4 *μ*g antigen (Fitzgerald Industries International, Acton, MA, USA). After washing, 20-time diluted samples were incubated with biotinylated detection antibody (chicken anti-human *α*1-microglobulin; ICL, Newberg, OR, USA). After washing, wells were incubated with HRP-conjugated streptavidin and binding was detected by measuring HRP activity using o-phenylenediamine as chromogenic substrate. Urinary *β*2-microglobulin (*β*2M) (Anogen, Mississauga, ON, Canada), heart-type fatty acid-binding protein (H-FABP) (Hytest, Turku, Finland), immunoglobulin G subclass 4 (IgG4), kidney injury molecule-1 (KIM-1), and neutrophil gelatinase-associated lipocalin (NGAL) (R&D Systems, Minneapolis, MN, USA) were measured by ELISA, as described previously [21]. Urinary* N*-acetyl-*β*-glucosaminidase (NAG) was measured using a modified enzyme assay according to Lockwood and Bosmann and corrected for nonspecific conversion (HaemoScan, Groningen, Netherlands) [22].

### 2.5. Preparation of FITC-Inulin Solution and Fluorescence Measurement

Prior to the animal experiments, 5% FITC-inulin (Sigma-Aldrich, Zwijndrecht, Netherlands) was dissolved in 0.9% NaCl by heating in boiling water and dialyzed to remove residual free FITC. The dialyzed FITC-inulin solution was sterilized by filtration through a 0.20 *μ*m syringe filter (Corning, New York, NY, USA).

Fluorescence of plasma and urine was measured within hours after collection in black 96-well plates (Fluotrac, Greiner Bio-One, Kremsmünster, Austria) in a FLUOstar Optima (BMG Labtech, Offenburg, Germany) at 485 nm excitation and 538 nm emission. Plasma and urine samples were buffered to pH 7.4 by dilution with HEPES 50 mmol/L pH 7.4 (5- and 10-fold, resp.) before fluorescence measurement. Matrix correction was applied for the standard curves.

### 2.6. Histology

For routine histological examination and scoring of tubular atrophy (TA), formalin-fixed, paraffin-embedded (FFPE) tissue sections were stained with periodic acid-Schiff (PAS). TA was defined as the presence of tubules with thickened tubular basement membranes and/or atrophic cells lining the tubules, with loss of brush border. TA was scored by two skilled observers in ten randomly selected cortical areas on ×100 magnification. The following semiquantitative scale was used: 0: no TA; 1: >0–10% TA (>0–10% of tubuli in the field shows atrophy); 2: 10–25% TA; 3: 25–50% TA; 4: 50–75% TA; 5: 75–100% TA. The average of ten fields was used for further statistical analysis. Photographs were taken on a Nikon Eclipse E800 microscope with a Nikon DXM1200 digital camera using the Nikon ACT-1 software version 2.70 (Nikon Netherlands, Lijnden, Netherlands).

### 2.7. *In Situ* Hybridization

Localization of CTGF mRNA was investigated as described in detail previously [23]. Briefly, 6 *μ*m thick FFPE tissue sections were dewaxed and rehydrated, incubated with proteinase K, postfixed, prehybridized, and hybridized with a 542-nt antisense CTGF DIG-labeled riboprobe for 16 h at 70°C. Upon washing, bound DIG was detected using alkaline phosphatase-conjugated sheep anti-DIG and NBT/BCIP (Roche, Almere, Netherlands). Incubation with sense DIG-labeled riboprobe was applied as negative control.

### 2.8. Quantitative Reverse Transcription PCR

For isolation of cortical mRNA, about 30 mg of the renal pole was cut using a scalpel. For medullary mRNA, fifteen 10 *μ*m cryosections per sample were put on a glass slide and medulla was identified based on location and morphology and microdissected using a scalpel. The remaining tissue was stained with hematoxylin and eosin to check the accuracy of medullary microdissection. Total RNA was isolated using the RNeasy RNA isolation kit (QIAGEN Benelux, Venlo, Netherlands). RNA was reverse-transcribed with SuperScript II Reverse Transcriptase (Invitrogen, Paisley, UK). Quantitative reverse transcription PCR (qRT-PCR) was performed on an Applied Biosystems 7900HT Fast Real-Time PCR System (Applied Biosystems, Nieuwerkerk aan den IJssel, Netherlands). Expression levels of* Ctgf* and the internal references,* Tbp* and* Gapdh,* were determined using Applied Biosystems inventoried Taqman Gene Expression Assays, containing primers and probe. Gene expression was quantified using the 2^−ΔΔCt^ method [24].

### 2.9. Calculations and Statistical Analysis

Data are presented as mean ± SD or median (interquartile range). Urinary CTGF, fractional CTGF excretion, CTGF mRNA (fold change), urinary markers, and human plasma CTGF data were logarithmically transformed to allow parametric analysis. Undetectable concentrations were set at half of the lowest detectable level. Urinary IgG4 was dichotomized as greater than or less than the detection limit because of a high proportion of subjects with undetectable levels. Differences were calculated using Student's* t*-test or Mann-Whitney *U* test where appropriate. Correlations between variables were evaluated by Pearson's and Spearman's correlation coefficients (*r* and *ρ*, resp.) where appropriate. Multiple linear regression analysis was performed to identify parameters independently associated with uCTGF. To explore the association between clusters of biomarkers and uCTGF, we used mean standard deviation scores (*Z*-scores), a method previously described by Schram et al. [25]. For each individual, the values of each marker were expressed as a *Z*-score, that is, (value in the individual minus the mean value in the study population) divided by the standard deviation. The proximal tubular reabsorption (PTR) *Z*-score was then calculated as (*Z*-score of *α*1M + *Z*-score of *β*2M)/2, the proximal tubular injury (PTI) *Z*-score as (*Z*-score of KIM-1 + *Z*-score of NAG + *Z*-score of NGAL)/3, and the combined proximal tubule (PT) *Z*-score as (PTR *Z*-score + PTI *Z*-score)/2. This approach was used in order to avoid underestimating the associations between the different markers and uCTGF. For all comparisons, a *P* value < 0.05 (two-tailed) was considered significant. The statistical analysis was performed using PASW Statistics software version 18.03 for Macintosh (SPSS Inc., Chicago, IL, USA) and GraphPad Prism software version 4.03 for Windows (GraphPad Software, San Diego, CA, USA).

In the diabetic mice study, urinary CTGF was expressed as 24-hour excretion rates. Urinary CTGF expressed per g creatinine provided similar results (see supplementary figures in the Supplementary Material available online at http://dx.doi.org/10.1155/2015/539787). Fractional excretion (FE) of CTGF was calculated as follows:

(1) $$\begin{array}{c} {FE}_{CTGF}=\frac{{\left[CTGF\right]}_{urine}\times {\left[inulin\right]}_{plasma}}{{\left[CTGF\right]}_{plasma}\times {\left[inulin\right]}_{urine}}\times 100\%. \end{array}$$

Assuming that rCTGF and endogenous CTGF (eCTGF) are handled similarly by the kidney and taking into account that rCTGF can only appear in urine by filtration from plasma whereby FE_rCTGF_ is only determined by CTGF filtered from the plasma (and not by intrarenal CTGF production), the relative contribution of plasma-derived eCTGF to urinary eCTGF could be estimated as follows:

(2) Fraction  of  ueCTGF  derived  from  plasma=FErCTGFFEeCTGF.

The relative contribution of intrarenally derived CTGF to urinary eCTGF could be estimated as follows:

(3) Fraction  of  ueCTGF  derived  from  a  local  intrarenal  source=1−FErCTGFFEeCTGF.

One diabetic mouse was excluded from this calculation because of unreliable measurements (below lower limit of quantification).

The absolute amount of urinary eCTGF derived from an intrarenal source was estimated as follows:

(4) Excretion  of  intrarenally-derived  eCTGF=excretion  of  eCTGF×1−FErCTGFFEeCTGF.

Renal clearance of FITC-inulin was calculated by the standard formula.

## 3. Results

### 3.1. Reduced Tubular Reabsorption Is a Major Determinant of Increased Urinary CTGF in Diabetic Mice

Diabetes was induced in C57BL/6 mice with STZ which caused pronounced hyperglycemia within one week. At the end of the study period the diabetic mice had developed DN, for example, increased albuminuria and decreased GFR (Table 1). Urinary excretion of CTGF was markedly elevated in all diabetic mice (999 fmol/24 h (190–2946), *P* < 0.0001) while in control mice uCTGF was measurable in only a minority, with a maximum of 57 fmol/24 h. Plasma CTGF was mildly increased in diabetic mice (340 pmol/L (290–370) versus 230 pmol/L (185–295) in control mice, *P* = 0.003).

**Figure 1:**
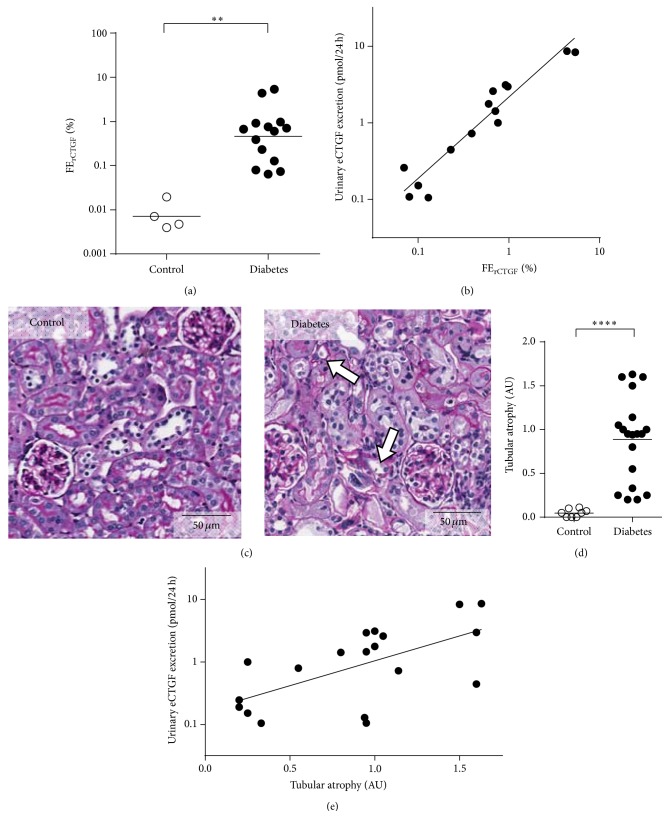
Urinary CTGF and tubular dysfunction in diabetic mice. (a) The fractional excretion of recombinant CTGF (FE_rCTGF_), a measure of tubular reabsorption failure, is increased in diabetes, ^*∗∗*^ *P* = 0.004 (Mann-Whitney *U* test). (b) Urinary endogenous CTGF excretion (eCTGF) correlates tightly with FE_rCTGF_, *r* = 0.95, *P* < 0.0001. (c) Tubular atrophy is not present in control mice but clearly visible in diabetic mice (white arrows). (d) Semiquantitative evaluation shows increased tubular atrophy in diabetic versus control mice, ^*∗∗∗∗*^ *P* < 0.0001 (Mann-Whitney *U* test). (e) Urinary eCTGF correlates with tubular atrophy, *r* = 0.62, *P* = 0.005.

To establish the influence of diabetic kidney disease on the renal handling of CTGF, we infused recombinant human CTGF (rCTGF) and FITC-inulin simultaneously by miniosmotic pumps. For detection of rCTGF levels we used an ELISA that does not cross-react with endogenous CTGF (eCTGF). This allowed us to study urinary excretion of plasma derived CTGF independent from intrarenally produced CTGF, since urinary recombinant CTGF is exclusively derived from filtered plasma rCTGF. On the contrary, urinary endogenous CTGF (ueCTGF) might also be derived from intrarenal production. Since CTGF is almost completely filtered from the plasma (sieving coefficient 0.74) [16] and rCTGF clearance and FITC-inulin clearance were not differentially affected between control and diabetic mice (*P* = 0.53, data not shown), fractional excretion of rCTGF (FE_rCTGF_) could be regarded as a measure of tubular CTGF passage with increased FE_rCTGF_ reflecting reduced tubular reabsorption of CTGF. We observed that in diabetic mice FE_rCTGF_ was strongly increased (72-fold, *P* = 0.004, Figure 1(a)), while in control mice tubular reabsorption of (filtered) rCTGF was virtually complete. FE_rCTGF_ showed a tight linear correlation with urinary excretion of endogenous CTGF (ueCTGF) in diabetic mice (*r* = 0.95, *P* < 0.0001, slope 1.1 on a logarithmic scale (Figure 1(b))) and emerged as an independent determinant of ueCTGF (*β* = 1.3, *P* < 0.001) in a multivariate model that included the following parameters: FE_rCTGF_, plasma CTGF, cortical and medullary CTGF gene expression (derived from qRT-PCR data of microdissected kidney, see below), and GFR. In addition to functional analysis of tubular reabsorption, histological analysis was performed. We observed no obvious glomerular damage in diabetic animals, but significant tubular atrophy was observed (Figures 1(c) and 1(d)), which correlated with ueCTGF (*r* = 0.62, *P* = 0.005, Figure 1(e)). This is consistent with tubular function as a major determinant of elevated ueCTGF.

### 3.2. In Diabetic Mice, a Large Part of uCTGF Is Intrarenally Derived

**Figure 2:**
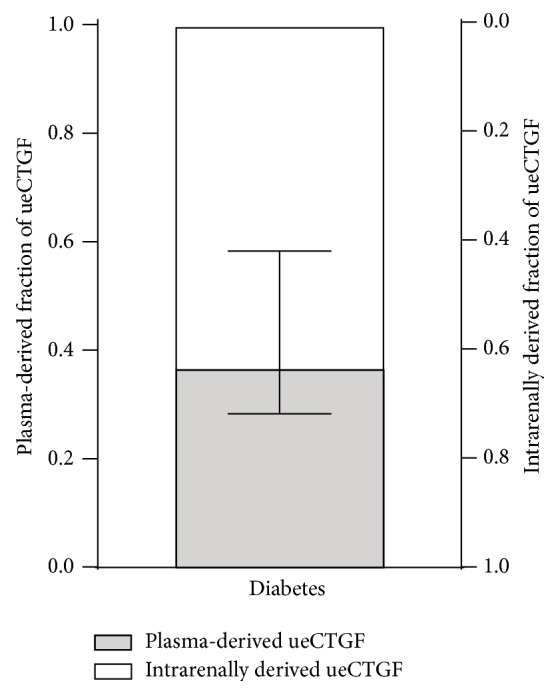
In diabetic mice, plasma-derived endogenous CTGF (eCTGF) accounts for 37% of urinary eCTGF (ueCTGF), while intrarenally derived CTGF accounts for 63% of ueCTGF (median with interquartile range).

To investigate the relative contribution of plasma-derived eCTGF to ueCTGF, we compared the fractional excretion (FE) of recombinant and endogenous CTGF. Assuming that rCTGF and eCTGF are handled similarly by the kidney, comparison of FE_rCTGF_ and FE_eCTGF_ allowed us to estimate the contribution of plasma-derived eCTGF to ueCTGF, the ratio FE_rCTGF_/FE_eCTGF_ representing the fraction of urinary eCTGF derived from plasma. This revealed that only 37 (29–55)% of urinary eCTGF could be accounted for by plasma-derived eCTGF, implying that most urinary CTGFs in diabetes must be derived from an intrarenal source and excreted into the tubular lumen (Figure 2). Due to the extremely low ueCTGF in healthy animals, the relative contribution of plasma-derived eCTGF to ueCTGF in the healthy situation could not be established.

### 3.3. CTGF Production Is Increased in Diabetic Kidney and Mainly Localized in Glomeruli and Medullary Tubules

**Figure 3:**
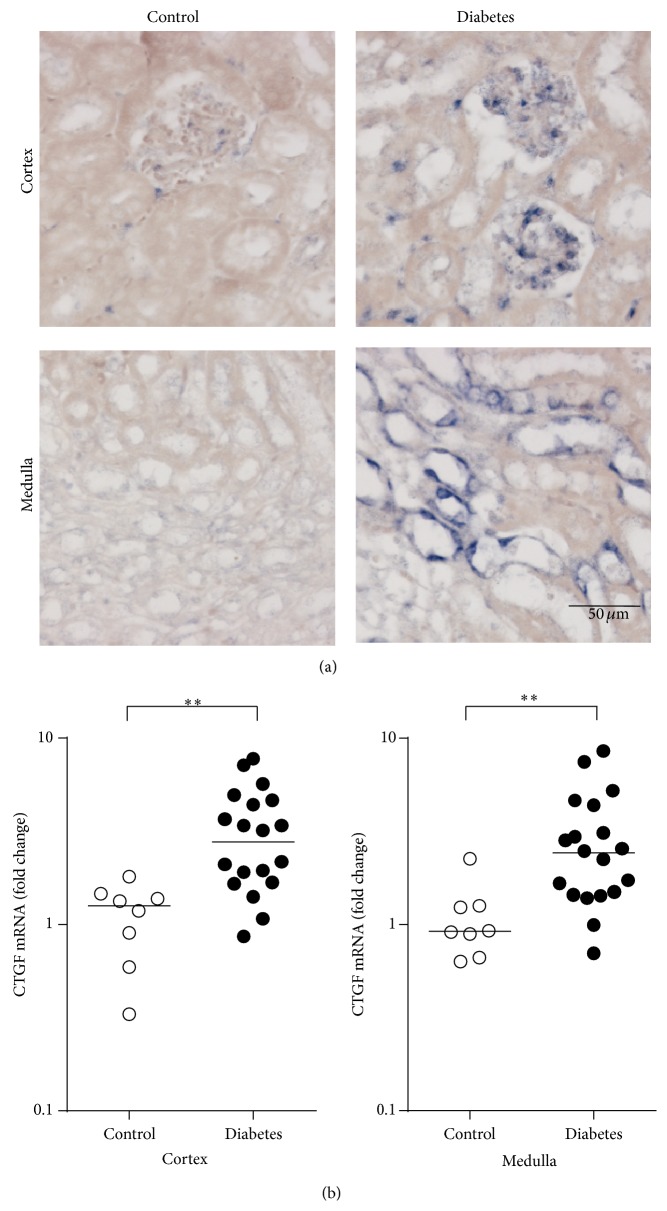
In diabetic kidneys, CTGF mRNA expression is increased in glomeruli and medullary tubules. (a)* In situ* hybridization of CTGF in control and diabetic mice, with little staining in control mice, and clear staining mainly in glomeruli and medullary tubules. (b) Quantitative RT-PCR of CTGF, showing increased levels in diabetic mice versus control mice, both ^*∗∗*^ *P* < 0.01 (Mann-Whitney *U* test).

To investigate the localization of intrarenal CTGF production, we performed CTGF* in situ* hybridization. This showed that CTGF expression was hardly detectable in control kidneys but abundant in diabetic kidneys, where it was mainly present in glomeruli and medullary tubules (Figure 3(a)). In agreement, qRT-PCR of microdissected kidney revealed that both cortical and medullary CTGF mRNA were significantly increased in diabetic animals compared with controls (2.5-fold and 2.7-fold, resp., both *P* < 0.01, Figure 3(b)). We did not observe differences in CTGF mRNA expression in cortex or medulla between animals infused with rCTGF or with vehicle, both in diabetic mice and in nondiabetic controls (data not shown).

### 3.4. Urinary Excretion of Intrarenally Produced CTGF Correlates with the Degree of Tubular Dysfunction

**Figure 4:**
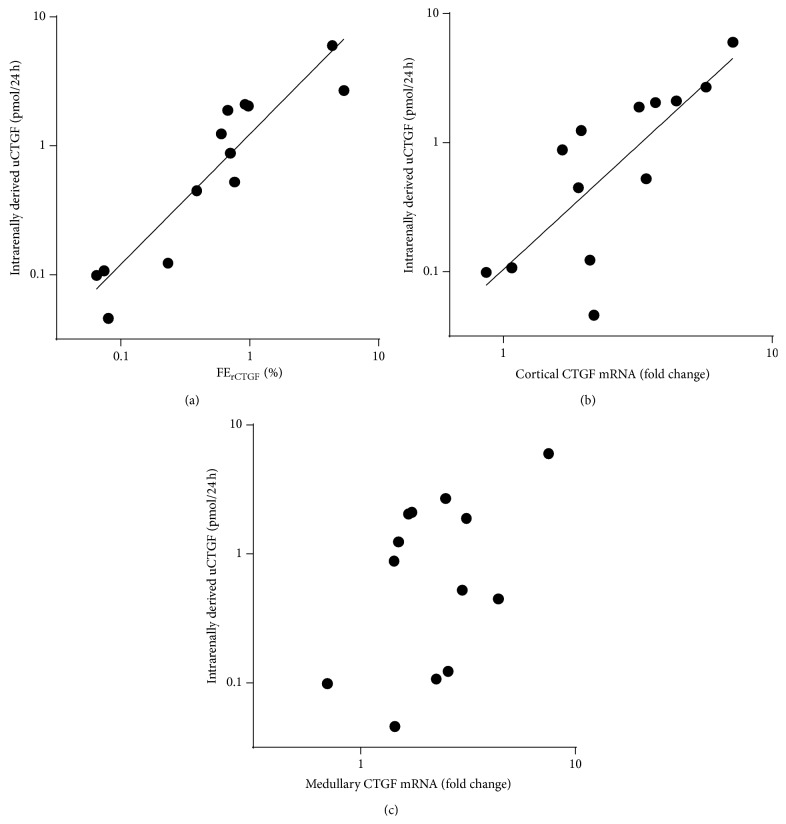
Urinary excretion of intrarenally produced CTGF correlates with the degree of tubular dysfunction. (a) Tight linear correlation between intrarenally derived uCTGF and FE_rCTGF_ (*r* = 0.92, *P* < 0.0001, slope = 1.0 ± 0.1) suggests that tubular reabsorption failure is a major determinant of intrarenally derived uCTGF. (b) Cortical CTGF mRNA levels correlate with the intrarenally derived urinary CTGF (uCTGF), *r* = 0.78, *P* = 0.002. (c) Medullary CTGF expression does not correlate with intrarenally derived uCTGF.

Intrarenally derived uCTGF showed a tight linear correlation with FE_rCTGF_ (*r* = 0.92, *P* < 0.0001, slope 1.0 on a logarithmic scale, Figure 4(a)). Cortical CTGF expression also correlated with intrarenally derived uCTGF (*r* = 0.78, *P* = 0.002, Figure 4(b)), while no such correlation was observed for medullary CTGF expression (*P* = 0.12, Figure 4(c)). In a multivariate model including cortical and medullary gene expression FE_rCTGF_ remained the only independent determinant of intrarenally derived uCTGF (*β* = 1.1, *P* < 0.001). These data suggest that tubular function is also an important determinant of intrarenally derived CTGF in the urine.

### 3.5. Increased uCTGF in Type 1 Diabetic Patients Is Independently Associated with Tubular Markers

To investigate the determinants of uCTGF in human diabetes, we studied the associations of uCTGF with tubular markers and a glomerular marker in a cohort of patients with diabetes mellitus type 1. The clinical characteristics of this patients study are shown in Table 2. IgG4 was used as marker for glomerular damage [26]. The low-molecular-weight proteins *β*2M and *α*1M were used as markers for reduced tubular reabsorption [27, 28]. KIM-1, NGAL, and NAG were used as markers for proximal tubular damage [29, 30] and H-FABP as a marker for distal tubular damage [31, 32]. In univariate analysis, uCTGF correlated with each of the damage markers (Table 3). In a multivariate linear regression model containing age, sex, eGFR, plasma CTGF, duration of diabetes, BMI, and HbA_1c_, each of the tubular markers remained independently associated with uCTGF while the glomerular damage marker IgG4 lost its significance (Table 3). When IgG4, the proximal tubular reabsorption, and proximal tubular injury *Z*-scores (or the combined proximal tubular *Z*-score) and H-FABP were included simultaneously into the model, both proximal and distal tubular markers, but not IgG4, were independently related to uCTGF (Table 3). These findings suggest that also in human diabetes uCTGF is dependent on tubular status, with elevated uCTGF reflecting both proximal tubular dysfunction and pathology in the distal tubule. Plasma CTGF also emerged as an independent determinant of uCTGF.

## 4. Discussion

Understanding the different determinants of elevated uCTGF in diabetes is essential for its proper interpretation as biomarker and pathogenic factor. Previously, we have shown that in the healthy kidney filtered CTGF is almost completely reabsorbed in the proximal tubules by megalin-mediated endocytosis and that impairment of tubular reabsorption results in increased urinary excretion of CTGF (in close correlation with that of *β*2M) [16]. Here we show that also in diabetes tubular damage is a major determinant of elevated uCTGF. Our findings indicate that in addition to reduced proximal reabsorption of uCTGF increased intrarenal CTGF production plays a role.

Both in human and in experimental diabetes, uCTGF was independently associated with decreased tubular reabsorption. While in experimental diabetes uCTGF correlated very tightly with tubular dysfunction, the association in human diabetes was somewhat less prominent. In addition, distal tubular damage appeared to play a role. In human diabetes there was a clear independent association between uCTGF and distal tubular damage marker H-FABP, suggesting increased CTGF secretion by the distal nephron. This conforms to the increased distal tubular CTGF mRNA expression that we observed in diabetic mice, although the correlation between uCTGF and medullary CTGF mRNA did not reach significance. Tubular damage might thus contribute to uCTGF in two ways, by (1) decreased proximal reabsorption of CTGF and (2) increased expression and luminal secretion of CTGF in the distal nephron. Possibly, solute overload to the distal nephron due to proximal tubular dysfunction may cause distal tubular injury and induce distal tubular CTGF expression. Although we are not aware of any clinical, experimental, or* in vitro* studies addressing CTGF expression specifically in medullary tubules in diabetic kidney disease, increased tubular CTGF protein expression has been reported before, both in human and in experimental diabetes, and was postulated to contribute to tubulointerstitial fibrosis via paracrine effects at the basolateral membrane [4, 33–35]. However, secretion of CTGF at the apical membrane of the distal tubular cell into the tubular lumen has not been described. It would be useful to have matched kidney biopsies from diabetic patients with known plasma and urine CTGF levels to explore the associations between increased uCTGF and pCTGF, tubular dysfunction, and local production of CTGF. Unfortunately, no matched human kidney biopsies were available in this study.

Although DN is traditionally viewed as a primarily glomerular disease, tubulointerstitial injury is also a major feature of DN with important prognostic significance [36, 37]. Tubular injury was shown to be an early event in the pathogenesis of DN and tubular proteinuria could identify patients susceptible to DN even earlier than albuminuria alone [37–41]. CTGF is upregulated early in DN [42]. In a nonhuman primate model of diabetes renal CTGF protein overexpression at 5-year duration of diabetes predicted 10-year albuminuria values, while at 5-year albuminuria values did not differ from nondiabetic controls [33]. Elevated uCTGF reflecting tubular damage might thus be a valuable prognostic marker in DN and useful for early detection of patients at risk. As compared to established markers of tubular damage, CTGF is of particular interest because it might also enhance tubulointerstitial fibrosis [4, 34, 35]. However, the true clinical value of uCTGF in diabetes still needs to be established.

In this study, we used a single high dose of STZ to induce diabetic kidney disease in mice. Since STZ has been associated with acute tubular injury [43], we cannot exclude that part of the tubular damage observed in our DN model might be directly related to STZ toxicity. However, at 10 weeks after infusion of STZ, we expect that the animals have recovered from the acute toxic effect of STZ. Instead, they have developed kidney disease in which both chronic glomerular and tubular damage are present, features that are also present in human diabetic kidney disease. To deduce the relative contributions of local production and tubular dysfunction in uCTGF, a DN model with both glomerular and tubular features was required. Since both features are not always present in non-STZ “pure” DN models, which typically manifest a primarily, if not exclusively, glomerular and sometimes vascular phenotype, we selected the STZ model for our studies.

In our mouse model of diabetic kidney disease, we observed that most uCTGF had a local renal source. In addition to increased medullary CTGF mRNA expression, CTGF production was increased in glomeruli, which is in agreement with previous reports [34, 35, 44–46]. However, it remains unclear how much intrarenally derived uCTGF has a glomerular source and how much is secreted at the apical membrane of the distal tubular cell. The tight independent linear association of intrarenally derived uCTGF with FE_rCTGF_ suggests that reduced tubular reabsorption of CTGF originating from a source upstream the proximal nephron, that is, the glomerulus, plays an important role.

In human diabetes, but not in experimental diabetes, plasma CTGF emerged as an independent determinant of uCTGF. This suggests that in human diabetes the amount of CTGF filtered from the plasma in the glomeruli also contributes to uCTGF, in addition to tubular damage. Plasma CTGF was shown to predict end-stage renal disease and mortality in macroalbuminuric patients [47]. For uCTGF this has not been investigated yet, but the independent association with plasma CTGF and tubular damage suggests promising biomarker value.

In conclusion, urinary CTGF excretion in diabetes is elevated as a result of both increased local production and reduced reabsorption due to tubular dysfunction. We submit that urinary CTGF may be a useful biomarker reflecting both glomerular and tubulointerstitial hallmarks of diabetic kidney disease.

## Supplementary Material

The Supplemental Material contains data of urinary CTGF expressed per g creatinine. Excretion of CTGF per creatinine gives comparable results as excretion expressed per 24-hour rates.

## Figures and Tables

**Table 1:** Characteristics of control mice and diabetic mice (parameters at termination of the study).

	Control	Diabetes
*N*	8	19
Plasma glucose (mmol/L)	11.6 (9.8–13.1)	21.5 (18.1–27.8)^a^
Body weight at start (g)	25.5 (24.7–26.9)	26.0 (24.9–27.0)
Body weight at termination (g)	25.6 (24.7–27.9)	22.1 (21.1–23.2)^a^
Kidney weight (mg)	150 (141–160)	139 (128–148)^a^
Kidney weight/body weight (mg/g)	5.7 (5.4–5.9)	6.3 (5.9–6.7)^a^
GFR (mL kg^−1^ min^−1^; inulin)	10.5 (8.4–12.4)	7.7 (6.3–9.4)^a^
AER (*μ*g/24 h)	135 (77–158)	400 (242–544)^a^
Plasma CTGF (pmol/l)	230 (185–295)	340 (290–370)^a^
Urinary CTGF (fmol/24 h)	≤57	999 (190–2946)^a^

Data are median (interquartile range); ^a^ *P* < 0.05 versus control mice in Mann-Whitney *U* test.

**Table 2:** General and clinicalcharacteristics of the diabetic patients.

	Diabetic patients
*N* (% man)	279 (46)
Normoalbuminuria (*N* (%))	142 (61)
Microalbuminuria (*N* (%))	64 (23)
Macroalbuminuria (*N* (%))	73 (26)
Age (years)	52 (41–62)
Duration of diabetes (years)	35 (26–42)
Body mass index (kg/m^2^)	24 (22–26)
HbA_1c_ (%)	8.4 (7.7–9.3)
Estimated GFR (mL min^−1^ 1.73 m^−2^)	75 (61–87)
Plasma CTGF (pmol/L)	136 (<127–270)
Urinary CTGF (pmol/g creatinine)	81 (54–118)

Data are median (interquartile range).

**Table 3:** Association of urinary CTGF with various damage markers in diabetic patients.

Damage marker	Model I		Model II		Model III		Model IV	
	*ρ*	*P *	Standard *β*	*P *	Standard *β*	*P *	Standard *β*	*P *
IgG4	0.155	0.009	0.106	0.063	−0.064	0.264	−0.058	0.302
*α*1M	0.378	<0.001	0.293	<0.001				
*β*2M	0.335	<0.001	0.226	<0.001				
KIM-1	0.226	<0.001	0.176	0.002				
NAG	0.166	0.005	0.145	0.012				
NGAL	0.235	<0.001	0.187	0.001				
H-FABP	0.371	<0.001	0.387	<0.001	0.258	<0.001	0.251	0.001
Proximal tubular reabsorption (PTR) *Z*-score					0.159	0.014		
Proximal tubular injury (PTI) *Z*-score					0.165	0.004		
Combined proximal tubule (PT) *Z*-score							0.266	<0.001
Plasma CTGF	0.330	<0.001	Variable^*∗*^	Variable^*∗∗*^	0.177	0.007	0.180	0.006

Model I: univariate (Spearman's *ρ*); model II includes age, sex, eGFR, duration of diabetes, BMI, HbA1c, plasma CTGF, and individual urinary marker; model III includes age, sex, eGFR, duration of diabetes, BMI, HbA1c, plasma CTGF, IgG4, PTR *Z*-score, PTI *Z*-score, and H-FABP; model IV includes age, sex, eGFR, duration of diabetes, BMI, HbA1c, plasma CTGF, IgG4, PT *Z*-score, and H-FABP. ^*∗*^Standard *β* varies from 0.175 to 0.256 and ^*∗∗*^ *P* value from <0.001 to 0.009, depending on the urinary marker included in the model.
