# Supplementary material for: Elevated Urinary Connective Tissue Growth Factor in Diabetic Nephropathy Is Caused by Local Production and Tubular Dysfunction
Source: J Diabetes Res. 2015 Jun 15;2015:539787. doi: 10.1155/2015/539787 (PMC4485941; doi:10.1155/2015/539787)
Supplement: Supplementary file 1 — The Supplemental Material contains data of urinary CTGF expressed per g creatinine. Excretion of CTGF per creatinine gives comparable results as excretion expressed per 24-hour rates. [file 539787.f1.pdf]

Electronic supplementary material

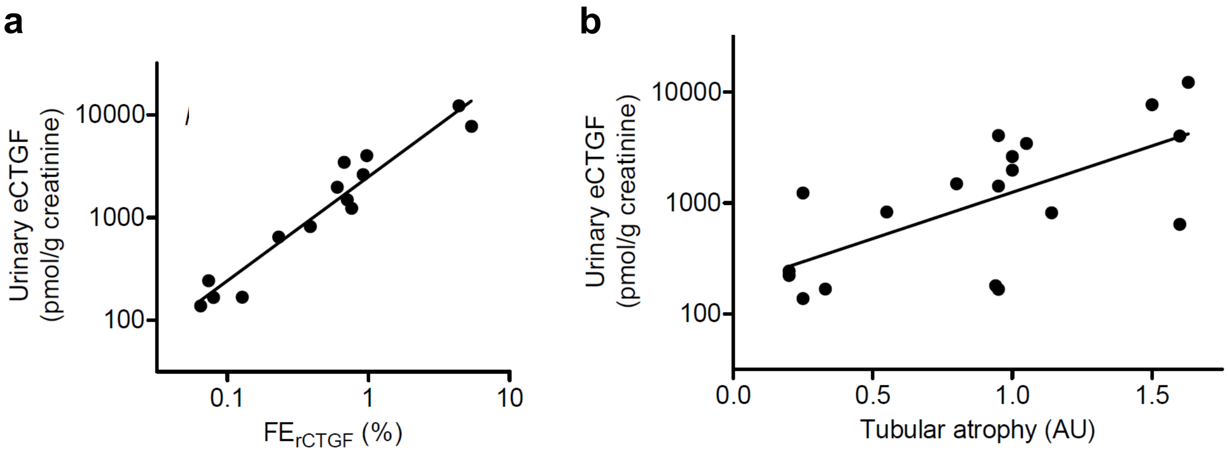

ESM Fig. 1 Excretion expressed per gram creatinine gives comparable results as excretion expressed per 24h (see Fig. 1 for results per 24h). (**a**) Urinary endogenous CTGF excretion (eCTGF) correlates tightly with FE_{rCTGF}, r=0.96, p<0.0001. (**b**) Urinary eCTGF correlates with tubular atrophy, r=0.67, p=0.002.

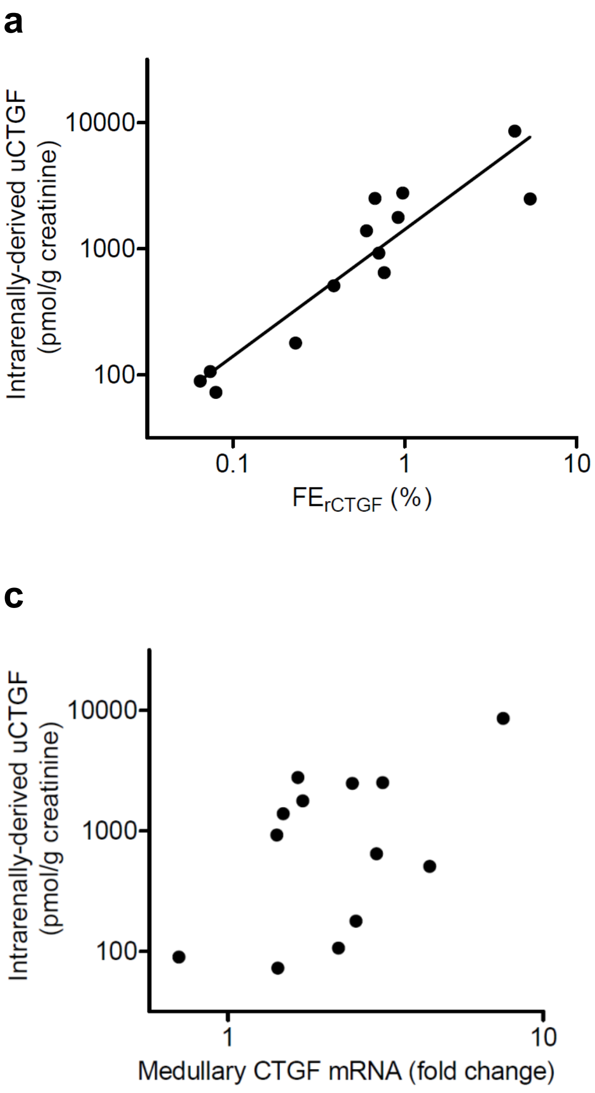

ESM Fig. 2 Excretion expressed per gram creatinine gives comparable results as excretion expressed per 24h (see Fig. 4 for results per 24h). (**a**) Tight linear correlation between intrarenally-derived uCTGF and FE_{rCTGF} (r=0.93, p<0.0001, *slope*=1.0±0.1) suggests that tubular reabsorption failure is a major determinant of intrarenally-derived uCTGF. (**b**) Cortical CTGF mRNA levels correlate significantly with the intrarenally-derived urinary CTGF (uCTGF), r=0.81, p=0.008. (**c**) Medullary CTGF expression does not correlate with intrarenally-derived uCTGF.
